# Chromosomal structures and repetitive sequences divergence in *Cucumis* species revealed by comparative cytogenetic mapping

**DOI:** 10.1186/s12864-015-1877-6

**Published:** 2015-09-25

**Authors:** Yunxia Zhang, Chunyan Cheng, Ji Li, Shuqiong Yang, Yunzhu Wang, Ziang Li, Jinfeng Chen, Qunfeng Lou

**Affiliations:** State Key Laboratory of Crop Genetics and Germplasm Enhancement, College of Horticulture, Nanjing Agricultural University, Nanjing, 210095 China

**Keywords:** *Cucumis*, Chromosome structure, Genomic in situ hybridization (GISH), Repetitive DNA, Evolution

## Abstract

**Background:**

Differentiation and copy number of repetitive sequences affect directly chromosome structure which contributes to reproductive isolation and speciation. Comparative cytogenetic mapping has been verified an efficient tool to elucidate the differentiation and distribution of repetitive sequences in genome. In present study, the distinct chromosomal structures of five *Cucumis* species were revealed through genomic in situ hybridization (GISH) technique and comparative cytogenetic mapping of major satellite repeats.

**Results:**

Chromosome structures of five *Cucumis* species were investigated using GISH and comparative mapping of specific satellites. Southern hybridization was employed to study the proliferation of satellites, whose structural characteristics were helpful for analyzing chromosome evolution. Preferential distribution of repetitive DNAs at the subtelomeric regions was found in *C. sativus*, *C hystrix* and *C. metuliferus*, while majority was positioned at the pericentromeric heterochromatin regions in *C. melo* and *C. anguria*. Further, comparative GISH (cGISH) through using genomic DNA of other species as probes revealed high homology of repeats between *C. sativus* and *C. hystrix*. Specific satellites including 45S rDNA, Type I/II, Type III, Type IV, CentM and telomeric repeat were then comparatively mapped in these species. Type I/II and Type IV produced bright signals at the subtelomeric regions of *C. sativus* and *C. hystrix* simultaneously, which might explain the significance of their amplification in the divergence of *Cucumis* subgenus from the ancient ancestor. Unique positioning of Type III and CentM only at the centromeric domains of *C. sativus* and *C. melo*, respectively, combining with unique southern bands, revealed rapid evolutionary patterns of centromeric DNA in *Cucumis*. Obvious interstitial telomeric repeats were observed in chromosomes 1 and 2 of *C. sativus*, which might provide evidence of the fusion hypothesis of chromosome evolution from x = 12 to x = 7 in *Cucumis* species. Besides, the significant correlation was found between gene density along chromosome and GISH band intensity in *C. sativus* and *C. melo*.

**Conclusions:**

In summary, comparative cytogenetic mapping of major satellites and GISH revealed the distinct differentiation of chromosome structure during species formation. The evolution of repetitive sequences was the main force for the divergence of *Cucumis* species from common ancestor.

**Electronic supplementary material:**

The online version of this article (doi:10.1186/s12864-015-1877-6) contains supplementary material, which is available to authorized users.

## Background

Plant genome contains large amount of repetitive sequences, even up to 85 % of genome in some plant species [1]. The distribution and copy number of repetitive DNA affect directly the genomic organization and chromosome structure through forming constitutive heterochromatin [2]. Tandem repetitive sequence (or called as satellite repeats) can yield species-specific patterns on chromosomes through fluorescence in situ hybridization (FISH) technology which could be used for karyotyping and phylogenetic analysis [2–5].

Chromosome structure differentiation is the main factor to affect the meiosis pairing which results to the reproductive obstacle among close relative species. The research about chromosome structure differentiation will provide key information for species formation and evolution. So far, it is still unpractical to investigate chromosome structure differentiation of most plant species based on the genome sequence due to the lack of genome information, especially majority of wild species. Genomic in situ hybridization (GISH) and comparative cytogenetic analysis provide powerful tools to study chromosome structure through the distribution pattern of characterized and uncharacterized gDNA sequences along chromosomes [2]. GISH technique has been widely applied for the analysis of parental genomes in hybrid and allopolyploids, and also the phylogenetics of closely species [2, 3, 6]. GISH-banding has also been proved to enable to elucidate genome-specific repeats having non-random distribution which were used for karyotype analysis [7–9]. The GISH banding pattern correlated with evolutionary distance and has been used to study the evolution of some species [2, 10].

**Fig. 1:**
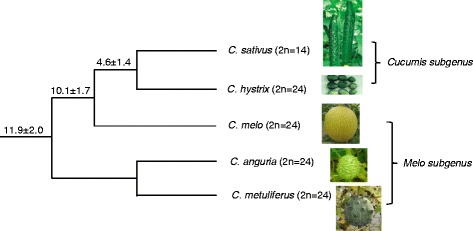
Phylogenetic relationship of the five *Cucumis* species used in comparative cytogenetic analysis. Values for age estimates (Mya) are placed above the branches

The genus *Cucumis* comprises of two subgenera, *Cucumis* and *Melo*, containing 52 species which include two economically important crops, *C. sativus* L. (cucumber, 2n = 2x = 14, 367 Mb) and *C. melo* L. (melon, 2n = 2x = 24, 450 Mb) [11]. Besides these two species, the species *C. anguria* (West Indian gherkin) and *C. metuliferus* (African horned cucumber) are commercially cultivated in some areas as well [12], and are also the important resources of resistance for some disastrous disease in cucumber and melon production, such as root knot nematode [13, 14]. In addition, many wild *Cucumis* species are of economic interest because of their potential useful genes for crop improvement. *C. hystrix* is the only wild species grouped as *Cucumis* subgenus together with *C. sativus* in *Cucumis* genus, while all others are classified into *Melo* subgenus (Fig. 1) [11]. Also, *C. hystrix* bearing multiple disease-resistant characteristics is the only wild species cross-compatible with *C. sativus* in this genus [15]. The researchabout the differentiation of chromosome structure will provide important information for the utilization of useful genes among *Cucumis* species through inter-specific hybridization. In genus *Cucumis*, cucumber is the only species which has the basic chromosome number of x = 7, while all other species have the chromosome number of x = 12, which provides interesting model for studying genome size and chromosome number evolution [11, 16]. However, except for cucumber and melon, little genomic information is available for the comparative genomic research among other *Cucumis* species. Comparative chromosome researches based on fluorescence in situ hybridization (FISH) of homologous repetitive sequences have been proved to be an efficient method for providing reliable information for elucidating the chromosome evolution among relative species [2, 17–19].

Specific repetitive sequences have been used to analyze their cytogenetic positions among relative species to reveal the chromosomal structural evolution [17, 18, 20]. In *Cucumis*, ribosomal DNAs have been comparatively mapped in *C. sativus* and *C. melo* to investigate their differentiation in which the unusual location of 45S rDNA interposed with centromeric domain provided evidence for the evolution of chromosomes 1 and 2 [21]. Except for rDNA sequences, in *Cucumis* species, a few satellites, such as Type I, Type II, Type III and Type IV from *C. sativus*, and CentM from *C. melo*, have been identified. Type I and Type II usually were grouped as Type I/II due to only one base different between them. Type I/II and Type IV were found to locate at the ends of almost all cucumber chromosomes [22, 23]. Type III and CentM repeats produced obvious signals at the primary constriction of cucumber and melon chromosome, respectively [22, 24]. Researches about these repeats mainly focus on their cytogenetic distribution in cucumber and melon. However, their cytogenetic distributions or homology in chromosomes among other *Cucumis* species remain to be investigated.

In this study, firstly self-GISH (sGISH) technology was applied to reveal the overall distribution of repetitive elements along chromosomes, through which the unique distribution patterns of repeats of each species were revealed. Secondly, comparative GISH (cGISH) was carried out to detect the homology of repeats among these species. Thirdly, specific repeats, like Type I/II, Type III and Type IV, combined with 45S rDNA and telomeric probes were used to investigate their distributions in these species. Southern hybridization was further employed to analyze the characteristics of genomic organization of specific repeats in different *Cucumis* species. Based on the results, the karyotypes including the differentiation of satellites among these species were analyzed, and the evolution of repetitive elements in *Cucumis* species was discussed as well.

## Results

### The distribution of repetitive sequences along chromosomes in *Cucumis* species revealed by self-GISH

**Fig. 2:**
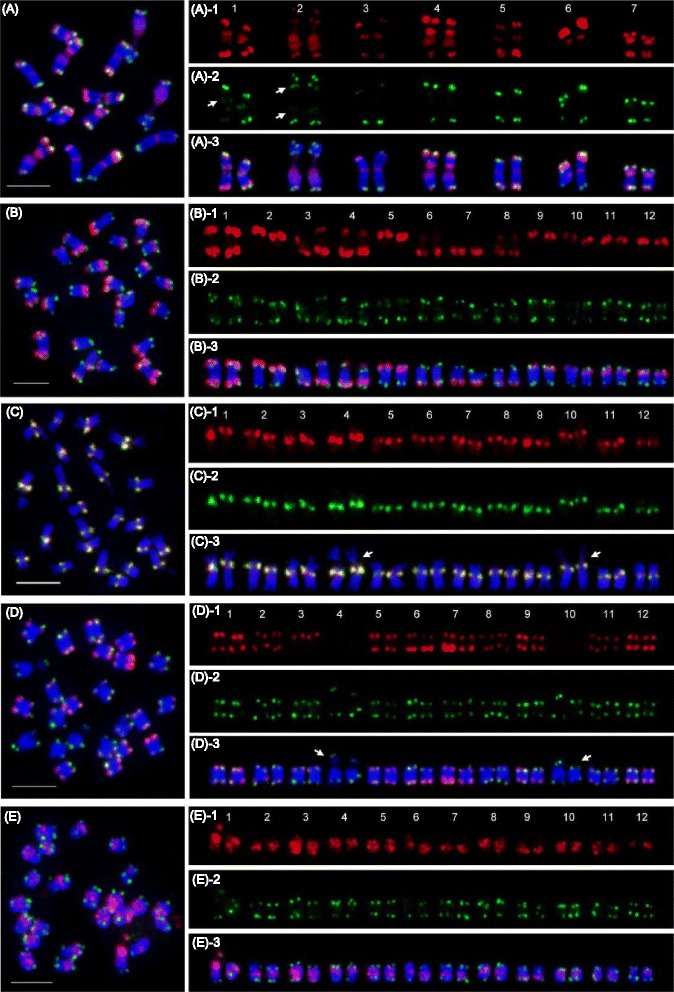
Distribution of repetitive sequences along chromosomes revealed by self-GISH in *Cucumis* species. **a** *C. sativus*; **b** *C. hystrix*; **c** *C. melo*; **d** *C. metuliferus*; **e** *C. anguria*. Red signals in (**a**), (**b**), (**c**), (**d**), and (**e**) represent the signals produced by genomic DNA themselves. Green signals in (**a**), (**b**), (**d**), and (**e**) represent the signals produced by *Arabidopsis* type telomere probes. Green signals in (**c**) represent the signals produced by *C. melo* centromere probe, CentM. Scale bars = 5 μm. Arrows in (**a**)-2 show interstitial telomere signals. *Arrows* in (**c**)-3 and (**d**)-3 show the nucleolar organizing regions (NORs)

The distribution patterns of repetitive DNAs along chromosomes in *Cucumis* species were investigated based on the sGISH method in which gDNA probe was hybridized onto its own metaphase chromosomes. Five *Cucumis* species including *C. sativus*, *C. hystrix*, *C. melo*, *C. metuliferus* and *C. anguria* which are of Asia or African origin (Fig. 1) were investigated [11]. The sGISH signal patterns for each species were showed in Fig. 2.

#### Signal patterns from C. sativus sGISH

Self-GISH produced unique signal patterns in each chromosome of *C. sativus* (Fig. 2a-1). Except for the end of long arm of chromosome 6, obvious signals were found at the ends of all other chromosomes. Very strong signals appeared at the both ends of chromosomes 1 and 7, short arm ends of chromosomes 4 and 6, and long arm end of chromosome 5. In addition, the distinct signal patterns were also observed at the pericentromeric heterochromatin regions of each chromosome, and very strong signals appeared in chromosomes 1, 2, 4 and 7. The signals in chromosomes 2 and 4 covered a large region across centromeres. The signal patterns revealed sGISH at the pericentromeric heterochromatin regions colocalized with those from 45S rDNA and Type III (Additional file 1: Figure S1).

**Fig. 3:**
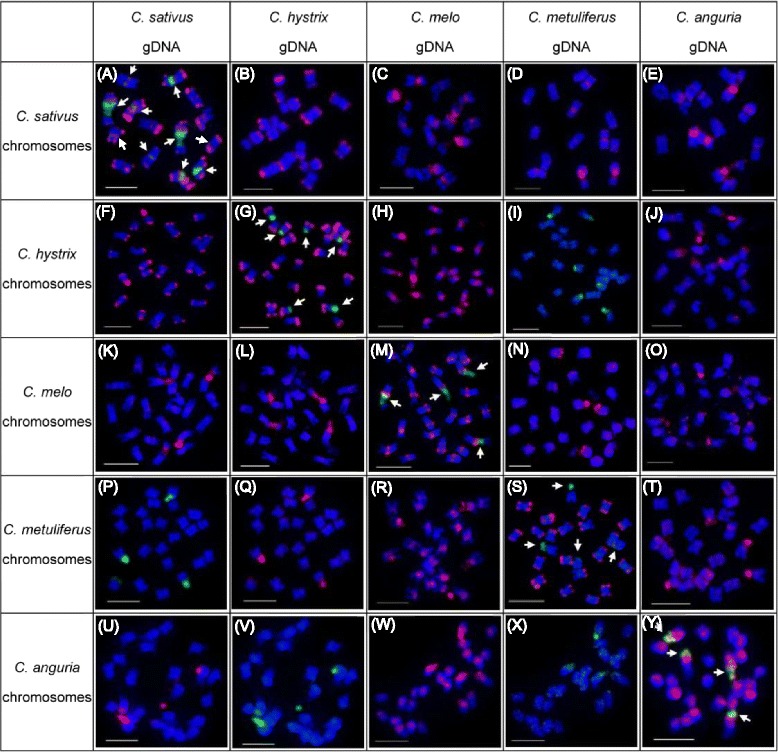
Distribution of conserved homologous sequences detected by reciprocal comparative GISH in *Cucumis* species. Each row represents the metaphase chromosomes from the same species, and the species was showed at left side; each column represents the signal patterns using total genomic DNA probe from one species, and the species used as probes was showed on the top. Green signals pointed by arrows in self-GISH pictures show the 45S rDNA loci. Scale bars = 5 μm

In order to confirm that the signals located on the ends of chromosomes are from other repetitive sequence rather than from telomeres, we probed simultaneously the *Arabidopsis* type telomere on *C. sativus* chromosomes. The telomere signals were detected at the ends of every chromosome of cucumber (green signals in Fig. 2a-2). The FISH from telomere and sGISH gave different signal patterns at the ends of each chromosome, which confirmed repeats-derived sGISH patterns (Fig. 2a-3). Interestingly, besides the terminal signals, some interstitial signals from telomere probe were also detected on chromosomes 1 and 2 (shown by arrows in Fig. 2a-2). Chromosome complement for *C. sativus* species was determined based on the signals of 45S rDNA (Fig. 3) and chromosome morphology according to previous reports [22, 25].

#### Signal patterns from C. hystrix sGISH

Due to no reference data about the karyotype of *C. hystrix* so far, the chromosome number was given here based on their sizes, in descending order (Fig. 2b). Self-GISH produced very strong signals at two ends or one end of chromosomes (Fig. 2b-1), which indicated a preferential location of repeats at the distal regions of chromosomes in this species. Chromosome 1 gave very strong signals at the both ends. Chromosomes 3, 4 and 8 display strong signals at the end of long arms, and weak signals at the end of short arms. Three weak signals produced in chromosomes 3, 4 and 8 were found to locate at the same positions with 45S rDNA loci as showed in Fig. 3g. All other chromosomes produced strong signal only at one end of chromosomes. We also compared the FISH mapping of telomere and sGISH. The FISH signals from telomere probe were detected at the ends of every chromosome and gave different patterns with sGISH, which indicted sGISH signals was produced by specific repeats rather than telomeres in *C. hystrix* (Fig. 2b-3).

#### Signal patterns from C. melo sGISH

For *C. melo*, signals from sGISH were detected at the primary constriction region of every chromosome (Fig. 2c-1). Further, we used CentM repeat located at the *C. melo* centromere regions [24], to compare the difference of sGISH and CentM signals. The FISH results showed that CentM produced exactly the same signal patterns as that from sGISH (Fig. 2c-2). In addition, we found that sGISH of this species did not produce any signals at the nucleolar organizing regions (NORs) (shown by arrow in Fig. 2c-3), which bear 45S rDNA loci (Fig. 3m). The FISH result from telomeric probe detected clear signals at the ends of each chromosome (Additional file 1: Figure S2). Unlike *C. sativus* and *C. hystrix*, sGISH of *C. melo* did not produce any signals at the ends of chromosomes.

#### Signal patterns from C. metuliferus sGISH

For *C. metuliferus*, except for two chromosomes without any sGISH signals, all other ten chromosomes possessed obvious sGISH signals. Among ten chromosomes having signals, nine chromosomes displayed signals at the both ends of chromosomes, while chromosome three produced signal only at one end (Fig. 2d-1). We also used the telomere probe to differentiate the signals of sGISH from telomeres. The different telomere signal pattern eliminated the possibility of sGISH signals from telomere (Fig. 2d-2). Like *C. melo* species, sGISH in *C. metuliferus* did not detected any signals at the nucleolar organizing regions (shown by arrow in Fig. 2d-3) which were detected by 45S rDNA probe (Fig. 3s). These results showed that the dominant repeats derived from GISH in *C. metuliferus* were located at the distal ends for majority of chromosomes.

#### Signal patterns from C. anguria sGISH

For *C. anguria*, sGISH detected the signals at the pericentromeric heterochromatin regions of all chromosomes (Fig. 2e-1). However, whether the signals are from the centromeric region or not could not be confirmed, because the centromere repeat sequence in this species is not available so far. Like in other species, the telomeric probe produced bright signals at the ends of each chromosome (Fig. 2e-2).

### The homology of repeats among *Cucumis* species based on the comparative genomic in situ hybridization

To reveal the homology of repeats among five *Cucumis* species, comparative GISH (cGISH) was employed to probe the signals of gDNA on metaphase chromosomes of all other species (Fig. 3). We also compared the FISH mapping of 45S rDNA on metaphase chromosomes of these species (shown by arrows in Fig. 3). The FISH signal patterns were shown in Fig. 3. cGISH from different xspecies revealed the distinct signal patterns when different gDNA probes were used. It is found that on *C. sativus* metaphase chromosomes, the gDNA of *C. hystrix* probed obvious subtelomere signals on majority of chromosomes (Fig. 3b), which is similar to the signal patterns produced by *C. sativus* sGISH at the chromosome ends (Figs. 2a and 3a). However, cGISH on *C. sativus* chromosomes using gDNAs of *C. melo*, *C. metuliferus* and *C. anguria* showed obvious signal patterns at the pericentromeric heterochromatin regions of some chromosomes (Fig. 3c, d, and e), which have same position with 45S loci (data not shown).

In *C. hystrix* metaphase chromosomes, cGISH using *C. sativus* gDNA probe produced bright signal patterns at majority of chromosome ends (Fig. 3f), which is similar to that produced by *C. hystrix* sGISH (Fig. 3g). Interestingly, gDNA of *C. melo* gave clear signals at the pericentromeric heterochromatin regions of all chromosomes of *C. hystrix*, and NORs as well (Fig. 3h). However, gDNAs of *C. metuliferus* and *C. anguria* did not probe strong signals along chromosomes except for the six 45S rDNA loci (Fig. 3i and j).

In *C. melo*, both gDNA probes of *C. sativus* and *C. hystrix* only detected four bright signals on four chromosomes (Fig. 3k and l), which were found to be the 45S rDNA loci (as shown with arrows in Fig. 3m). The signal patterns detected by gDNA of *C. metuliferus* and *C. anguria* showed bright 45S rDNA loci signals, as well as some weak telomeric and scattered signals along every chromosome (Fig. 3n and o).

Similar to *C. melo*, in *C. metuliferus* metaphase chromosomes, gDNA probes from *C. sativus* and *C. hystrix* only produced signals at the NORs (Fig. 3p and q). Genomic DNA probe from *C. melo* detected the pericentromeric heterochromatin regions of every chromosome, and NORs as well (Fig. 3r). However, cGISH using *C. anguria* gDNA produced weak scattered signals on some chromosomes, and clear NORs signals.

In *C. anguria* chromosome spreads, gDNA probes of *C. sativus* and *C. hystrix* also only detected the 45S rDNA loci (Fig. 3u and v). cGISH using gDNA probe of *C. melo* produced clear signals around pericentromeric heterochromatin domains, though the signal intensity varied in different chromosomes, and 45S rDNA loci (Fig. 3w). Genomic DNA probe of *C. metuliferus* produced scattered signals along *C. anguria* chromosomes (Fig. 3x).

Different signal pattern of 45S rDNA were produced among five *Cucumis* species. In *C. sativus*, 10 chromosomes displayed 45S rDNA loci, including 6 very strong loci and 4 weak signals, which all were located adjacent to centromeric regions. Six chromosomes in *C. hystrix* bear 45S rDNA loci which were mapped at the distal of chromosomes. And only 4 chromosomes displayed 45S rDNA loci with interstitial or subtelomeric locations in *C. melo*, *C. metuliferus* and *C. anguria* species (shown by arrows in Fig. 3). However, all five species bore a pair of 5S loci (data not shown).

### Comparative mapping of specific satellites revealed the significant divergence among *Cucumis* species

**Fig. 4:**
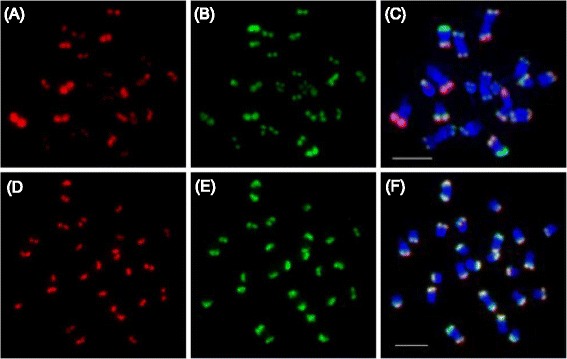
FISH mapping of two types of satellites on metaphase chromosomes of *C. sativus* and *C. hystrix.* **a** Signals of Type I/II on *C. sativus*. **b** Signals of Type IV on *C. sativus*. **c** Merged picture from (**a**) and (**b**). (**d**) Signals of Type I/II on *C. hystrix*. **e** Signals of Type IV on *C. hystrix*.** f** Merged picture from (**d**) and (**e**). Scale bars = 5 μm

High similarity of signal patterns shown by cGISH from *C. satvius* and *C. hystrix* (shown by Fig. 3a, b, f and g) is likely to be explained by the high homology of satellites. To further reveal the homology of satellites between species, specific types of satellites, including Type I/II, Type III, Type IV and CentM were comparatively mapped on metaphase chromosomes of five *Cucumis* species. Among these satellites, Type I/II and Type IV have been mapped on the subtelomeric domains of *C. sativus* chromosomes, and Type III was located at the centromeric regions of *C. sativus* chromosomes [22, 23, 26], and CentM was identified as the centromeric satellite DNA of melon [27]. As expected, Type I/II (Fig. 4a) and Type IV (Fig. 4b) produced bright signals at the subtelomeric regions of almost all chromosomes, and Type I/II was mapped at the distal position compared with Type IV in *C. sativus* chromosome spreads (Fig. 4c). Interestingly, Type I/II and Type IV also produced clear signals at the end of chromosomes of *C. hystrix* (Fig. 4d, e and f), presenting the similar relative positions as them in *C. sativus* chromosomes, though their copy numbers varied significantly in individual chromosome based on the sizes and intensities of the FISH signals. However, Type I/II and Type IV did not probe clear signal in other three *Cucumis* species. Type III and CentM probes only detected the centromeric signals in *C. sativus* and *C. melo*, respectively, and no signal in any other species (Data not shown).

**Fig. 5:**
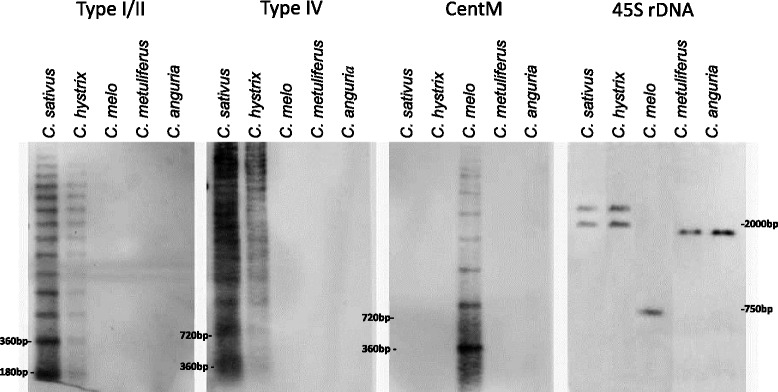
Southern hybridization of specific satellites. Genomic DNA of each lane stands for five *Cucumis* species, *C. sativus*, *C. hystrix*, *C. melo*, *C. metuliferus* and *C. anguria*, respectively. Each blot was probed separately with satellites Type I/II, Type IV, CentM, and 45S rDNA

To characterize the molecular organization and the abundance of the specific tandem repeats observed by fluorescence in situ hybridization, southern blot was conducted to further analyze the genomic organization of them in *Cucumis* species. Genomic DNA of *C. sativus*, *C. hystrix*, *C. melo*, *C. metuliferus* and *C. anguria* was digested with the restriction enzymes *EcoR*I, blotted, and hybridized with Type I/II, Type IV, CentM and 45S rDNA probes. We found that *C. sativus* and *C. hystrix* shared almost the same patterns of Type I/II and Type IV, but the fragments from *C. sativus* are stronger than those from *C. hystrix*. The ladder of fragments indicates a tandem organization of these repeats. However, no bands appeared in other three species for Type I/II and Type IV probes. For CentM, obvious ladder of bands was observed only in *C. melo* species. For 45S rDNA, five species gave three types of patterns. *C. sativus* and *C. hystrix* produced the same two bands, and *C. metuliferus* and *C. anguria* shared the same one band. *C. melo* produced one band with shorter size compared with that from other species (Fig. 5).

### The relationship between GISH patterns and gene distribution

**Fig. 6:**
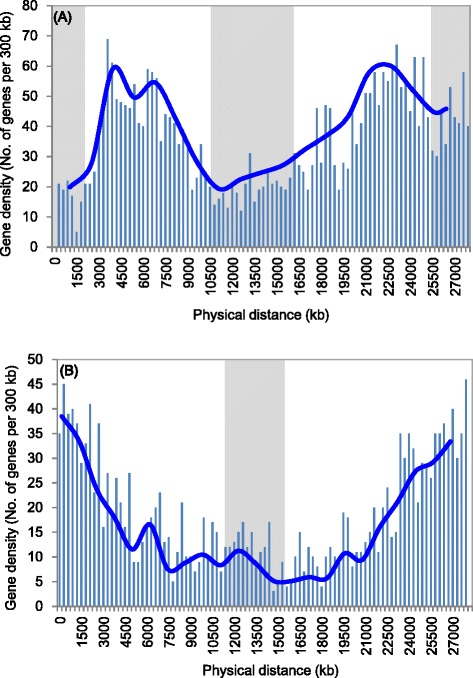
Distribution of gene density along the chromosome 5 of cucumber (**a**) and chromosome five of melon (**b**). The blue line show the overall trend of gene density along chromosome, and the gray shadow parts show the rough heterochromatin region according to FISH results. The “physical distance” on x-axis means the end to end distance (from telomere to telomere)

The sGISH showed the distinct signal patterns in different *Cucumis* species. Some species such as *C. sativus*, *C. hystrix* and *C. metuliferus* produced bright telomeric signals, and some species, such as *C. melo* and *C. anguria* only detected signals in pericentromeric heterochromatin region. These results showed the obvious difference of chromosomal structure in *Cucumis* species. We further analyzed the gene density along chromosome to reveal the relationship between chromosomal structure and gene distribution. In *Cucumis*, *C. sativus* and *C. melo* are the only two species whose genome sequences are available [28, 29]. The chromosome 5 was selected randomly to conduct this analysis. According to the Cucumber Genome Database (http://cucumber.genomics.org.cn/page/cucumber/index.jsp) and Melon Genome Database (https://melonomics.net/), the numbers of annotated genes along chromosome 5 in two species are 3344 and 1840, respectively. The number of annotated genes per 300 kb was calculated to investigate the gene density along chromosome 5 of both species. The distributions of gene density along the two chromosomes were illustrated in Fig. 6a and b, respectively. The uneven distribution of gene density was observed along chromosomes 5 in both species. In general, lowest gene density was observed in the heterochromatin regions (showed by the gray color in Fig. 6) in two chromosomes, and relative high gene density in the euchromatin region of chromosome arms. However, obvious different distribution patterns were found between two chromosomes. In *C. sativus* chromosome 5, the highest gene density appeared at the central regions of chromosome arms, and decreased towards the ends of chromosome and pericentromeric heterochromatin region (Fig. 6a). However, in *C. melo* chromosome 5, the gene density increased towards the ends of chromosome, and the highest appeared at the ends (Fig. 6b). The distribution patterns of gene density of these two chromosomes were found to be relative with the sGISH patterns. The regions with strong sGISH signals, like the ends of chromosomes and pericentromeric heterochromatin regions in the chromosome 5 of *C. sativus* (Fig. 2a-1) have the lowest gene density (Fig. 6a). Similarly, in chromosome 5 of *C. melo* species, the lowest gene density (Fig. 6b) was detected at the pericentromeric heterochromatin region with strong sGISH signals. Meanwhile, a significant increase of gene density towards the ends of chromosome arms was observed where nearly no sGISH signals were detected (Fig. 2c-1).

## Discussion

### The chromosome structural differentiation in *Cucumis* species detected by GISH

In *Cucumis* species, only the genome sequences of *C. sativus* and *C. melo* are available so far. The research of evolution relationship based on the genome sequence among them and other majority of wild species with unavailable genome information is impossible now. Further, even in the era of high throughout sequencing, there are missing gaps caused by technical inability to correctly sequence tandem repeats and to accurately determine their copy number and chromosome position. GISH and FISH provide powerful tools for elucidating the genome organization and chromosome structure. Based on the sGISH in present study, we found that five *Cucumis* species produced different bands that showed the unique chromosome structures of each species. In general, *C. sativus*, *C. hystrix*, and *C. metuliferus* detected significant sGISH bands at the ends of chromosomes, whereas other two species, especially *C. melo* did not produce any signals at the ends of chromosomes. This is likely to be explained by the lack of repeats at the distal end of chromosomes in *C. melo* species. In our previous investigation for chromatin structures, obvious heterochromatin knob (usually rich in repetitive sequences) was found at the chromosome ends of *C. sativus* [26], while no obvious heterochromatin region existed on *C. melo* chromosome ends [30]. Thus, results from sGISH in this study are likely to directly reflect the chromosomal structure about repeats distribution in *Cucumis* species.

The high similar signal patterns detected by cGISH between *C. sativus* and *C. hystrix* indicated the high repeats homology in subtelomeric regions in *C. sativus* and *C. hystrix* (Fig. 3a, b, f, and g). Genomic DNA probes of *C. sativus* and *C. hystrix* detected mainly 45S rDNA loci in other three *Cucumis* species which demonstrated the low repeats homology between *C. sativus*/*C. hystrix* and other three species.

### Chromosomal rearrangements during *Cucumis* species formation inferred from the distribution of satellites

*C. sativus* is the only species with x = 7 chromosomes in *Cucumis*, and all other species in this genus have x = 12 chromosomes. So far, there are two hypotheses about the evolution of chromosome numbers in *Cucumis* species. Early studies supported the hypothesis that the species with x = 7 chromosomes gave rise to species with x = 12 chromosomes through chromosomal fission events [31], while more recent researches supported another hypothesis that x = 7 species was formed by chromosomal fusion from x = 12 progenitor species [11, 32, 33]. Molecular and cytogenetic data from current study proposed that chromosomes 1 and 2 of *C. sativus* appeared to be the result of fusion of two chromosomes of x = 12 species [16, 21]. Interstitial telomeric repeats were proposed to be a relic of two ancestral chromosomes fusion in some eukaryotes [34–36]. Interstitial telomeric signals detected in chromosomes 1 and 2 of *C. sativus* in this study are likely to be the vestige of the chromosome fusion of two progenitor species. This phenomenon conversely also supported the fusion hypothesis for chromosome evolution in *Cucumis* species.

**Fig. 7:**
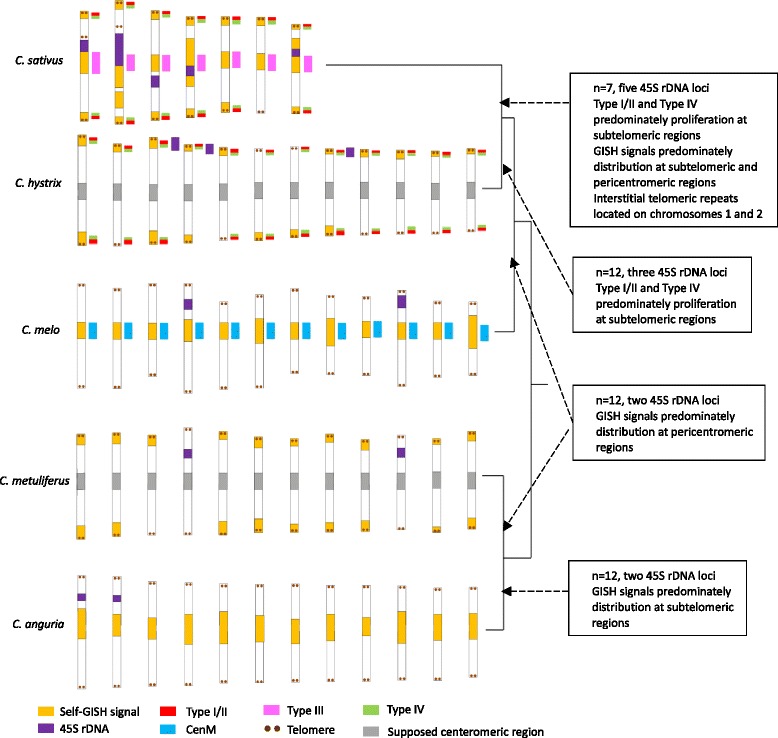
Idiograms of five *Cucumis* species (*left*) and information of GISH signals and satellite repeats distribution (*right*)

The accumulation of specific repeats has been proved to be the main force of species formation. Comparative FISH revealed that *C. sativus* and *C. hystrix* shared high homology of satellites, and Type I/II and Type IV produced strong signals on the chromosomes of both species, which was also confirmed by the southern hybridization results (Figs. 4 and 5). Among all *Cucumis* species, *C. sativus* and *C. hystrix* are the only two species grouped as *Cucumis* subgenus, while all other are classified into *Melo* subgenus. *C. hystrix* is also the only species which is cross-compatible with *C. sativus* in this genus [37, 38], and had the closest relationship with *C. sativus* (Fig. 1) [11]. Our results might indicate that the preferential proliferation of specific repeats, like Type I/II and Type IV diverged the *Cucumis* and *Melo* subgenera from common ancestor species (Fig. 7), which happened within approximately ten million years [11]. This phenomenon was also found in other species, such as *Nicotiana* in which new satellite repeats evolved and amplified within about 5 million years [39].

Genomic DNA probe of *C. melo* produced obvious pericentromeric heterochromatin signals in all tested *Cucumis* species which likely indicated relative conserved repeat type in this region in *C. melo* species. However, specific repeat from *C. melo*, CentM did not detect obvious FISH signal in other species, also no visible southern blot bands in other four species (Fig. 5). Hence, the signals from *C. melo* gDNA probe in pericentromeric regions of other species might be derived from other specific unknown repeats rather than CentM.

In addition, gDNA probes of *C. metuliferus* and *C. anguria* did not detected clear signals in other species except for 45S rDNA loci which implied the high divergence between these two species and the others. Evidence from plastid DNA sequences and nuclear markers also grouped *C. metuliferus* and *C. anguria* as of Africa and other three species as of Asian origin, and diverged from the common ancestry about 12 million years ago [11].

We also found that the number of 45S rDNA loci have the significant difference in two subgenera, *Melo* and *Cucumis*, though all species bear a pair of 5S rDNA loci (data now shown). All three species in subgenus *Melo* displayed two pairs of 45S rDNA loci, while subgenus *Cucumis* had more 45S rDNA loci. *C. hystrix* and *C. sativus* displayed six and ten loci of 45S rDNA, respectively. Therefore, the expansion of ribosomal DNA loci is likely to be contributed to the divergence of subgenus *Cucumis* from the common ancestry (Fig. 7). The southern results revealed that *C. sativus* and *C. hystrix* shared the same 45S rDNA band pattern, and *C. metuliferus* and *C. anguria* shared another same band pattern, while *C. melo* had the different southern blot pattern. This result might explain the phylogenetic relationship among five species, which is coincidence with the report from the analysis of sequence (Fig. 1) [11]. However, researches from majority of plants showed that in spite of the wide dispersion capacity of ribosomal DNA, the number of rDNA locus tends to be restricted in two and four per diploid karyotype [40]. The significant meaning of more 45S rDNA loci in subgenus *Cucumis* than that in subgenus *Melo* species remains to be explored.

## Conclusion

Significant differentiation of chromosome structures was observed among *Cucumis* species, which could be revealed by the repeats distribution along chromosomes. The preferential accumulation of repeats mainly around subtelomeric regions was found in *C. sativus*, *C. hystrix* and *C. metuliferus* species, while in *C. melo* and *C. anguria* species, majority of repeats located at the pericentromeric regions. *C. sativus* and *C. hystrix* species shared the high homology of satellites, like Type I/II and Type IV which were positioned on the subtelomeric domains. Besides, expansion of these specific satellites was supposed to diverge the *Cucumis* subgenus from ancient ancestor. What’s more, the copy number and nucleotide sequence are the main changing parameters during the evolution process in the specific repeats. Among *Cucumis* species, except for *C. sativus* and *C. melo*, nearly no genomic resources are available in all other species. As a consequence, the chromosome structures shown by GISH and comparative FISH of satellites in this study provide an important clue and strike out path for elucidating the formation of species and evolution in *Cucumis* species.

## Methods

### Plant materials

Five species with different geographical origin and basic chromosome numbers in *Cucumis* were used for this study. Phylogenetic relationship of the five *Cucumis* species was showed in Fig. 1 [11]. These species belong to two subgenera of the genus of *Cucumis*, respectively. *C. sativus* and *C. hystrix* are from subgenus *Cucumis*, originated in Asia. Species of *C. anguria* and *C. metuliferus* belong to subgenus *Melo*, with of African origin. *C. melo* is believed to be of Asian origin which belongs to subgenus *Melo* [11].

### Chromosome preparation

The procedure for mitotic chromosomes preparation was essentially the same as published protocols [26] with some modifications. The seeds of all species were geminated on moistened filter paper at 28 °C. Lateral roots were induced through cutting away the main root tips. About 1 cm long lateral root tips were harvested and pretreated in 0.002 M 8-hydroxyquinoline at room temperature for 2 h, then fixed in 3:1 Carnoy’s fixative solution for at least 1 day. For chromosome preparation, the fixed root tips were digested with enzyme mixtures containing 4 % cellulase R-10 (Yakult, http://www.yakult.co.jp) and 2 % pectinase (Sigma-Aldrich, http://www.sigmaaldrich.com) in 1× PBS buffer, pH 5.5, at 37 °C for 50 min, followed by replacing the enzyme solution with deionized water and keeping it on ice for about 10 min. The digested root tips were then fixed in 3:1 Carnoy’s fixative solution. The slides with well-spread metaphase chromosomes were obtained according to the published protocol [41].

### DNA probes

For GISH, total genomic DNA (gDNA) was extracted from young leaves of seedlings using the cetyltrimethylammonium bromide (CTAB) based method described by Murray and Thompson [42]. DNA quality was evaluated by electrophoresis in 1 % agarose gel. DNA concentration was estimated using the ultraviolet spectrophotometer. About 1 ug gDNA of each species was used for probe labeling. For repeat DNA probes, Type III repeat of with the size of 177 bp (GenBank accession no. 18287), and CentM repeat of melon with the size of 352 bp (GenBank accession no. 3929695) were used for identification of centromeres of *C. sativus* and *C. melo*, respectively. Plasmids pTa71 with the size of 9 kb from wheat [43] was used to detect the 45S ribosomal DNAs. Type I/II repeat with the size of 182 bp (GenBank accession no. 18285) and Type IV repeat with the size of 360 bp (GenBank accession no. 18288) were also used for comparative mapping. The *Arabidopsis*-type telomere DNA was generated by polymerase chain reaction method in the absence of template using primers (TTTAGGG)_4_ and (CCCTAAA)_4_ according to Ijdo et al. [44]. The gDNA and all repeat DNA probes were labeled with either biotin-dUTP or digoxigenin-dUTP (Roche, http://www.roche-applied-science.com) by standard nick translation reaction.

### Fluorescence in situ hybridization

FISH was carried out essentially according to published procedures [45]. Twenty microliter hybridization mixture containing denaturated probes, 50 % formamide and 10 % dextrane sulfate in 2 × SSC were applied to the denaturated slide. Slide was incubated at 37 °C overnight. Signals were detected using a fluorescein isothiocyanate-conjugated antibiotin antibody and a rhodamine-conjugated anti-digoxigenin antibody (Roche, http://www.roche-applied-science.com), respectively. Images were captured using a SENSYS (http://www.photometrics.com) CCD camera attached to an Olympus (http://www.olympus-global.com) BX51 microscope. The CCD camera was controlled using FISH view 5.5 software (Applied Spectral Imaging Inc, http://www.spectral-imaging.com). Images were processed using Adobe Photoshop 5.0 (Adobe Systems, http://www.adobe.com).

### Southern hybridization

Ten microgram of gDNA of five species were digested with restriction enzymes (with recognition sites for six bases) *EcoR*I (Takara, Japan), separated by electrophoresis at 25 V (1 V/cm) overnight on a 0.7 % agarose gel in 0.5× TBE buffer. Specific satellite DNAs, including 45S rDNA, Type I/II, Type III, Type IV and CentM, were labeled to be probes following the standard protocols according to the procedures of DIG High Prime DNA Labeling and Detection Starter Kit II (Roche, Germany). DNA fragments were transferred onto nylon membranes (Roche, Germany) by electric transfer (Tanon EPS 200, China). Hybridization was carried out with 25 ng/ml of chosen digoxigenin-labeled probe at 42 °C in oven (HL-2000 HybriLinker, UVP) for 20 h. A stringency washes were conducted by using 0.5× SSC (Solution of Sodium Citrate) containing 0.1 % SDS (sodium dodecylsulphate), and the kit working solutions for appropriate time. The hybridization signals were visualized on recorded using Tanon 5200 (Tanon, China).

### Gene density analysis

To analyze the relationship between chromosomal structure and the distribution of genes, the numbers of annotated genes per 300 kb along chromosomes of *C. sativus* and *C. melo* were calculated. The distribution patterns of gene density along chromosomes were plotted. The annotated gene location and number were accessed through Cucumber Genome Database homepage (http://cucumber.genomics.org.cn/page/cucumber/index.jsp) and Melon Genome Database homepage (https://melonomics.net/).

## Additional file

Additional file 1: Figure S1. FISH mapping of 45S rDNA and Type III on *C. sativus* metaphase chromosomes. (A) 45S rDNA signals. (B) Type III signals. (C) Merged picture. Scale bars = 5 μm. **Figure S2.** FISH mapping of Telomere on *C. melo* metaphase chromosomes. Scale bars = 5 μm. (PDF 159 kb)

## Abbreviations

FISH: Fluorescence in situ hybridization
GISH: Genomic in situ hybridization
sGISH: Self-genomic in situ hybridization
cGISH: Comparative GISH

## References

[1] Schnable PS, Ware D, Fulton RS, Stein JC, Wei F, Pasternak S (2009). The B73 maize genome: complexity, diversity, and dynamics. Science.

[2] Markova M, Vyskot B (2009). New horizons of genomic in situ hybridization. Cytogenet Genome Res.

[3] Jiang J, Gill BS (2006). Current status and the future of fluorescence in situ hybridization (FISH) in plant genome research. Genome.

[4] Markova M, Michu E, Vyskot B, Janousek B, Zluvova J (2007). An interspecific hybrid as a tool to study phylogenetic relationships in plants using the GISH technique. Chromosome Res.

[5] Raskina O, Barber JC, Nevo E, Belyayev A (2008). Repetitive DNA and chromosomal rearrangements: speciation-related events in plant genomes. Cytogenet Genome Res.

[6] Ellneskog-Staam P, Salomon B, von Bothmer R, Anamthawat-Jonsson K (2003). The genome composition of hexaploid Psammopyrum athericum and octoploid *Psammopyrum pungens* (*Poaceae*: *Triticeae*). Genome.

[7] Belyayev A, Raskina O, Nevo E (2001). Evolutionary dynamics and chromosomal distribution of repetitive sequences on chromosomes of *Aegilops speltoides* revealed by genomic *in situ* hybridization. Heredity (Edinb).

[8] Zhou JP, Yang ZJ, Li GR, Liu C, Ren ZL (2008). Discrimination of repetitive sequences polymorphism in *Secale cereale* by genomic *in situ* hybridization-banding. J Integr Plant Biol.

[9] She C, Liu J, Diao Y, Hu Z, Song Y (2007). The distribution of repetitive DNAs along chromosomes in plants revealed by self-genomic *in situ* hybridization. J Genet Genomics.

[10] Lim KY, Kovarik A, Matyasek R, Chase MW, Clarkson JJ, Grandbastien MA (2007). Sequence of events leading to near-complete genome turnover in allopolyploid *Nicotiana* within five million years. New Phytol.

[11] Sebastian P, Schaefer H, Telford IR, Renner SS (2010). Cucumber (*Cucumis sativus*) and melon (*C. melo*) have numerous wild relatives in Asia and Australia, and the sister species of melon is from Australia. Proc Natl Acad Sci U S A.

[12] Garcia-Mas J, Monforte AJ, Arus P (2004). Phylogenetic relationships among *Cucumis* species based on the ribosomal internal transcribed spacer sequence and microsatellite markers. Plant Sys Evol.

[13] Nugent PE, Dukes PD (1997). Root-knot nematode resistance in *Cucumis* species. HortScience.

[14] Winstead NN, Sasser JN (1956). Reaction of cucumber varieties to five root-knotnematodes (*Meloidogynespp*.). Plant Dis Rep.

[15] Chen JF, Staub J, Adelberg J, Lewis S, Kunkle B (2002). Synthesis and preliminary characterization of a new species (amphidiploid) in *Cucumis*. Euphytica.

[16] Yang L, Koo DH, Li D, Zhang T, Jiang J, Luan F (2014). Next-generation sequencing, FISH mapping, and synteny-based modeling reveal mechanisms of decreasing dysploidy in *Cucumis*. Plant J.

[17] Lim KY, Kovarik A, Matyasek R, Chase MW, Knapp S, McCarthy E (2006). Comparative genomics and repetitive sequence divergence in the species of diploid *Nicotiana* section Alatae. Plant J.

[18] Rosato M, Galian JA, Rossello JA (2012). Amplification, contraction and genomic spread of a satellite DNA family (E180) in *Medicago* (*Fabaceae*) and allied genera. Ann Bot.

[19] Cioffi MB, Martins C, Bertollo LA (2009). Comparative chromosome mapping of repetitive sequences. Implications for genomic evolution in the fish, *Hoplias malabaricus*. BMC Genet.

[20] Grzywacz B, Chobanov DP, Maryanska-Nadachowska A, Karamysheva TV, Heller KG, Warchalowska-Sliwa E (2014). A comparative study of genome organization and inferences for the systematics of two large bushcricket genera of the tribe *Barbitistini* (*Orthoptera*: *Tettigoniidae*: *Phaneropterinae*). BMC Evol Biol.

[21] Koo DH, Nam YW, Choi D, Bang JW, de Jong H, Hur Y (2010). Molecular cytogenetic mapping of *Cucumis sativus* and *C. melo* using highly repetitive DNA sequences. Chromosome Res.

[22] Zhao X, Lu J, Zhang Z, Hu J, Huang S, Jin W (2011). Comparison of the distribution of the repetitive DNA sequences in three variants of *Cucumis sativus* reveals their phylogenetic relationships. J Genet Genomics.

[23] Han YH, Zhang ZH, Liu JH, Lu JY, Huang SW, Jin WW (2008). Distribution of the tandem repeat sequences and karyotyping in cucumber (*Cucumis sativus* L.) by fluorescence *in situ* hybridization. Cytogenet Genome Res.

[24] Han Y, Zhang Z, Liu C, Liu J, Huang S, Jiang J (2009). Centromere repositioning in cucurbit species: implication of the genomic impact from centromere activation and inactivation. Proc Natl Acad Sci U S A.

[25] Koo DH, Choi HW, Cho J, Hur Y, Bang JW (2005). A high-resolution karyotype of cucumber (*Cucumis sativus* L. 'Winter Long') revealed by C-banding, pachytene analysis, and RAPD-aided fluorescence *in situ* hybridization. Genome.

[26] Lou Q, He Y, Cheng C, Zhang Z, Li J, Huang S (2013). Integration of high-resolution physical and genetic map reveals differential recombination frequency between chromosomes and the genome assembling quality in cucumber. PloS One.

[27] Liu C, Liu J, Li H, Zhang Z, Han Y, Huang S (2010). Karyotyping in melon (*Cucumis melo* L.) by cross-species fosmid fluorescence *in situ* hybridization. Cytogenet Genome Res.

[28] Huang S, Li R, Zhang Z, Li L, Gu X, Fan W (2009). The genome of the cucumber, *Cucumis sativus* L. Nat Genet.

[29] Garcia-Mas J, Benjak A, Sanseverino W, Bourgeois M, Mir G, Gonzalez VM (2012). The genome of melon (*Cucumis melo* L.). Proc Natl Acad Sci U S A.

[30] Lou Q, Zhang Y, He Y, Li J, Jia L, Cheng C (2014). Single-copy gene-based chromosome painting in cucumber and its application for chromosome rearrangement analysis in *Cucumis*. Plant J.

[31] Bhaduri PN, Bose PC (1947). Cyto-genetical investigations in some common cucurbits, with special reference to fragmentation of chromosomes as a physical basis of speciation. J Genet.

[32] Ghebretinsae A, Thulin M, Barber JC (2007). Relationships of cucumbers and melons unraveled: molecular phylogenetics of cucumis and related genera (*benincaseae*, *cucurbitaceae*). Am J Bot.

[33] Renner SS, Schaefer H, Kocyan A (2007). Phylogenetics of *Cucumis* (*Cucurbitaceae*): cucumber (*C. sativus*) belongs in an Asian/Australian clade far from melon (*C. melo*). BMC Evol Biol.

[34] Ijdo JW, Baldini A, Ward DC, Reeders ST, Wells RA (1991). Origin of human chromosome 2: an ancestral telomere-telomere fusion. Proc Natl Acad Sci U S A.

[35] Uchida W, Matsunaga S, Sugiyama R, Kawano S (2002). Interstitial telomere-like repeats in the *Arabidopsis thaliana* genome. Genes Genet Sys.

[36] Azzalin CM, Nergadze SG, Giulotto E (2001). Human intrachromosomal telomeric-like repeats: sequence organization and mechanisms of origin. Chromosoma.

[37] Chen JF, Adelberg J (2000). Interspecific hybridization in *Cucumis* - Progress, problems, and perspectives. HortScience.

[38] Chen JF, Staub JE, Tashiro Y, Isshiki S, Miyazaki S (1997). Successful interspecific hybridization between *Cucumis sativus* L. and *C. C-hystrix* Chakr. Euphytica.

[39] Koukalova B, Moraes AP, Renny-Byfield S, Matyasek R, Leitch AR, Kovarik A (2010). Fall and rise of satellite repeats in allopolyploids of *Nicotiana* over c. 5 million years. New Phytol.

[40] Roa F, Guerra M (2012). Distribution of 45S rDNA sites in chromosomes of plants: structural and evolutionary implications. BMC Evol Biol.

[41] Iovene M, Wielgus SM, Simon PW, Buell CR, Jiang J (2008). Chromatin structure and physical mapping of chromosome 6 of potato and comparative analyses with tomato. Genetics.

[42] Murray MG, Thompson WF (1980). Rapid isolation of high molecular weight plant DNA. Nucleic Acids Res.

[43] Gerlach WL, Bedbrook JR (1979). Cloning and characterization of ribosomal RNA genes from wheat and barley. Nucleic Acids Res.

[44] Ijdo JW, Wells RA, Baldini A, Reeders ST (1991). Improved telomere detection using a telomere repeat probe (TTAGGG)n generated by PCR. Nucleic Acids Res.

[45] Lou Q, Iovene M, Spooner DM, Buell CR, Jiang J (2010). Evolution of chromosome 6 of *Solanum* species revealed by comparative fluorescence *in situ* hybridization mapping. Chromosoma.

