# Supplementary material for: Chromosomal structures and repetitive sequences divergence in Cucumis species revealed by comparative cytogenetic mapping
Source: BMC Genomics. 2015 Sep 25;16:730. doi: 10.1186/s12864-015-1877-6 (PMC4583154; doi:10.1186/s12864-015-1877-6)
Supplement: Additional file 1: Figure S1. — FISH mapping of 45S rDNA and Type III on C. sativus metaphase chromosomes. (A) 45S rDNA signals. (B) Type III signals. (C) Merged picture. Scale bars = 5 μm. Figure S2. FISH mapping of Telomere on C. melo metaphase chromosomes. Scale bars = 5 μm. (PDF 159 kb) [file 12864_2015_1877_MOESM1_ESM.pdf]

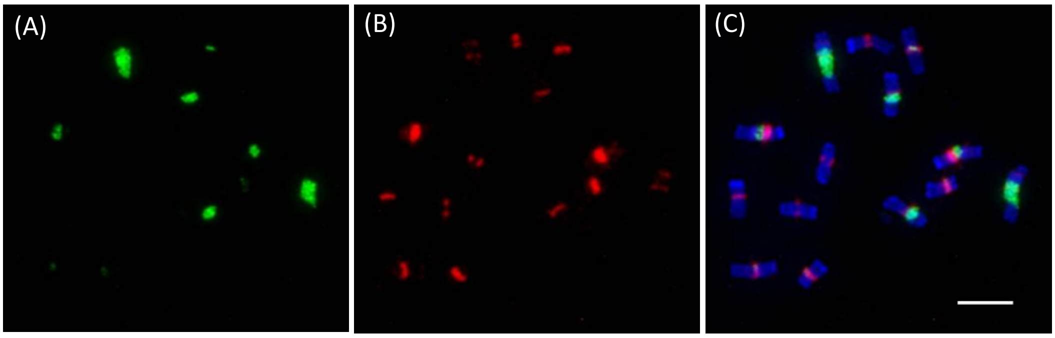

Figure S1 FISH mapping of 45S rDNA and Type III on *C. sativus* metaphase chromosomes. (A) 45S rDNA signals. **(B)** Type III signals. **(C)** Merged picture. Scale bars = 5 μm.

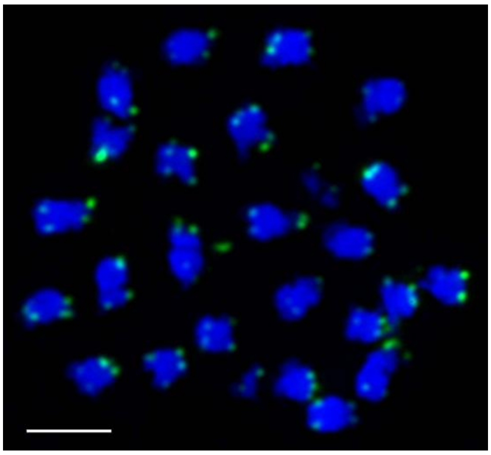

Figure S2 FISH mapping of Telomere on *C. melo* metaphase chromosomes. Scale bars = 5 μm.
